# Sono‐Activatable Semiconducting Polymer Nanoreshapers Multiply Remodel Tumor Microenvironment for Potent Immunotherapy of Orthotopic Pancreatic Cancer

**DOI:** 10.1002/advs.202305150

**Published:** 2023-10-23

**Authors:** Meng Li, Yue Liu, Yijing Zhang, Ningyue Yu, Jingchao Li

**Affiliations:** ^1^ State Key Laboratory for Modification of Chemical Fibers and Polymer Materials College of Biological Science and Medical Engineering Donghua University Shanghai 201620 China

**Keywords:** immunotherapy, orthotopic pancreatic cancer, polymer nanoparticles, sonodynamic therapy, tumor microenvironment

## Abstract

Due to the complicated tumor microenvironment that compromises the efficacies of various therapies, the effective treatment of pancreatic cancer remains a big challenge. Sono‐activatable semiconducting polymer nanoreshapers (SPN_DN_H) are constructed to multiply remodel tumor microenvironment of orthotopic pancreatic cancer for potent immunotherapy. SPN_DN_H contain a semiconducting polymer, hydrogen sulfide (H_2_S) donor, and indoleamine 2,3‐dioxygenase (IDO) inhibitor (NLG919), which are encapsulated by singlet oxygen (^1^O_2_)‐responsive shells with modification of hyaluronidase (HAase). After accumulation in orthotopic pancreatic tumor sites, SPN_DN_H degrade the major content of tumor microenvironment hyaluronic acid to promote nanoparticle enrichment and immune cell infiltration, and also release H_2_S to relieve tumor hypoxia via inhibiting mitochondrion functions. Moreover, the relieved hypoxia enables amplified sonodynamic therapy (SDT) under ultrasound (US) irradiation with generation of ^1^O_2_, which leads to immunogenic cell death (ICD) and destruction of ^1^O_2_‐responsive components to realize sono‐activatable NLG919 release for reversing IDO‐based immunosuppression. Through such a multiple remodeling mechanism, a potent antitumor immunological effect is triggered after SPN_DN_H‐based treatment. Therefore, the growths of orthotopic pancreatic tumors in mouse models are almost inhibited and tumor metastases are effectively restricted. This study offers a sono‐activatable nanoplatform to multiply remodel tumor microenvironment for effective and precise immunotherapy of deep‐tissue orthotopic tumors.

## Graphical abstract

A sono‐activatable semiconducting polymer nanoreshaper is reported to precisely deliver immunotherapeutic drugs and multiply remodel tumor microenvironment for boosting immunological effect in orthotopic pancreatic cancer. The nanoreshaper remodels extracellular matrix stroma, hypoxia, and indoleamine 2,3‐dioxygenase‐based immunosuppressive pathway. Such a multiple remodeling strategy enables a forceful antitumor immunological effect to effectively inhibit growth of deep‐tissue orthotopic pancreatic tumors and tumor metastases.

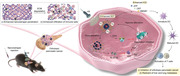

## 1 Introduction

Pancreatic cancer is a type of digestive malignant tumor with five‐year survivals rate less than 8%.^[^ ^1^ ^]^ Surgery is the main option for treatments of pancreatic cancer, while it is difficult for resection of advanced‐stage tumors.^[^ ^2^ ^]^ Adjuvant chemotherapy and/or radiotherapy after resection has been considered to improve the therapeutic potency and prolong patient survival.^[^ ^3^ ^]^ However, pancreatic cancer often shows chemoresistance and radiotherapy resistance that obviously compromise the therapeutic outcomes, and frequent treatments will increase the toxic effects and adverse events.^[^ ^4^ ^]^ In addition, pancreatic cancer shows high possibilities of tumor cell spreads and tumor metastases that cannot be effectively handled by chemotherapy and radiotherapy.^[^ ^5^ ^]^ Therefore, it is an urgent need to explore alternative strategies for pancreatic cancer treatment.

Immunotherapy is a novelty therapeutic strategy for cancer because it can eliminate the primary tumors, and restrict spread of tumor cells and relapse of remaining tumor cells.^[^ ^6^ ^]^ As one of the major methods of immunotherapy, immune checkpoint blockade therapy has achieved remarkable therapeutic outcomes in a variety of tumor types.^[^ ^7^ ^]^ Nevertheless, the immunotherapeutic benefits for pancreatic cancer are poor because of the highly immunosuppressive tumor microenvironment.^[^ ^8^ ^]^ Some traditional therapies, such as chemotherapy and photodynamic therapy have been used to drive immunogenic cell death (ICD), thereby enhancing the outcomes of immunotherapy.^[^ ^9^ ^]^ However, the shallow tissue penetration of photodynamic therapy and severe side effects of chemotherapy still limit their applications for pancreatic cancer treatments.^[^ ^10^ ^]^ Sonodynamic therapy (SDT) is another therapeutic strategy involving the generation of reactive oxygen species (ROS) that triggers ICD of tumor cells for boosting the immune responses.^[^ ^11^ ^]^ SDT can precisely focus the therapeutic actions on tumor sites to greatly improve the selectivity and specificity, thus overcoming the toxicity concerns of chemotherapy.^[^ ^12^ ^]^ Moreover, SDT breaks shallow depth limitations of photodynamic therapy and is able to treat deep‐tissue tumors.^[^ ^13^ ^]^ The combination of SDT with immunotherapy provides an attractive strategy for treatments of deep‐seated orthotopic pancreatic cancer.^[^ ^14^ ^]^

Complicated tumor microenvironment is the main characteristic of pancreatic cancer, which also plays key roles in attenuating the therapeutic outcomes of various therapies.^[^ ^15^ ^]^ Pancreatic cancer secretes extremely rich extracellular matrix (ECM) in tumor microenvironment that builds a compact physical barrier to hinder drug diffusions and penetrations and T cell infiltrations into tumors.^[^ ^16^ ^]^ The extremely hypoxic conditions will limit the potency of oxygen‐dependent therapies, such as SDT.^[^ ^17^ ^]^ The up‐regulation of immune checkpoint signaling in pancreatic cancer tumor microenvironment contributes to failure of immunotherapy.^[^ ^9a^ ^]^ Although various stroma‐targeting strategies are utilized to remodel tumor microenvironment for maximizing the therapeutic potencies of pancreatic cancer, the benefits are still dissatisfactory.^[^ ^18^ ^]^ Multiple remodeling of tumor microenvironment is necessary to further reinforce treatment efficacy of pancreatic cancer, which however has not been explored.

**Figure 1:**
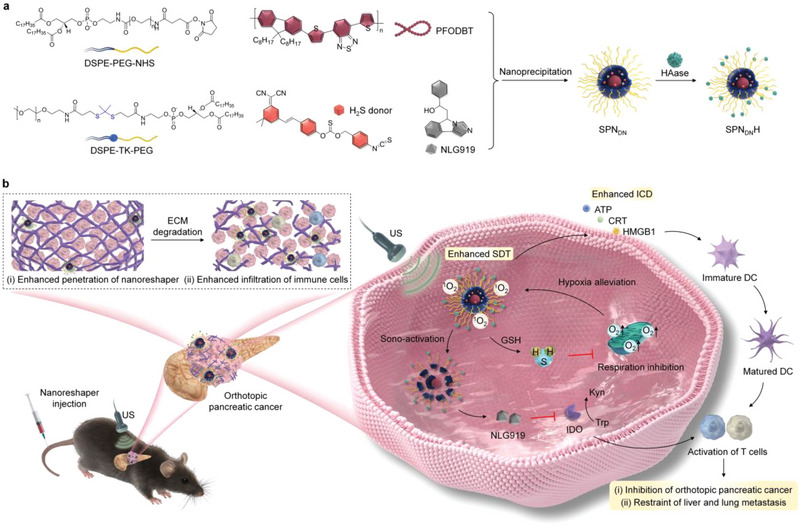
Design of SPN_DN_H to multiply remodel tumor microenvironment for potent immunotherapy of orthotopic pancreatic cancer. a) Illustration of PFODBT, H_2_S donor, and NLG919 for construction of SPN_DN_H. b) Schematic illustration of treatment of orthotopic pancreatic cancer through multiply remodeling of tumor microenvironment.

We herein report semiconducting polymer nanoreshapers (SPN_DN_H) to multiply remodel tumor microenvironment for potent immunotherapy. Orthotopic pancreatic tumor models were used because of the abundant hyaluronic acid in tumor ECM.^[^ ^16c^ ^]^ To achieve controlled release of immunotherapeutic drug, SPN_DN_H are designed to contain singlet oxygen (^1^O_2_)‐responsive components with encapsulations of a semiconducting polymer (PFODBT), a hydrogen sulfide (H_2_S) donor, and NLG919 in nanoparticle core and surface modification of hyaluronidase (HAase) (**Figure** **1**a). Due to HAase‐mediated degradation of hyaluronic acid in tumor microenvironment, enrichment of nanoparticles and infiltration of cytotoxic T lymphocytes in orthotopic pancreatic tumor sites are improved (Figure 1b). SPN_DN_H‐based H_2_S release inhibits cell respiration to relieve tumor hypoxia, thus further amplifying SDT effect. The generated ^1^O_2_ during SDT triggers ICD and destroys ^1^O_2_‐responsive shells for on‐demand delivery of NLG919 to inactivate immunosuppressive indoleamine 2,3‐dioxygenase (IDO). As such, dense ECM stroma, hypoxia and immunosuppressive pathways in tumor microenvironment are multiply remodeled, leading to a high antitumor effect via combining SDT with immunotherapy. This therapeutic strategy is demonstrated to almost completely inhibit the growth of orthotopic pancreatic tumors and abolish tumor metastases.

## 2 Results and Discussion

### 2.1 Sonodynamic and Drug Release Properties of Nanoreshapers

H_2_S donor was synthesized as reported in one of our previous works (Figure S1, Supporting Information).^[^ ^19^ ^]^ H_2_S donor and NLG919 loaded semiconducting polymer nanoparticles (SPN_DN_) were formed through nanoprecipitation of H_2_S donor, NLG919 and semiconducting polymer (PFODBT) at a feeding weight ratio of 1:1:2 using DSPE‐PEG‐NHS and further DSPE‐TK‐PEG as the amphiphilic polymers. HAase was then conjugated onto the surface of SPN_DN_ to allow the formation of nanoreshapers (SPN_DN_H). Single H_2_S donor loaded semiconducting polymer nanoparticles (SPN_D_) were also prepared via nanoprecipitation and used as another control counterpart.

**Figure 2:**
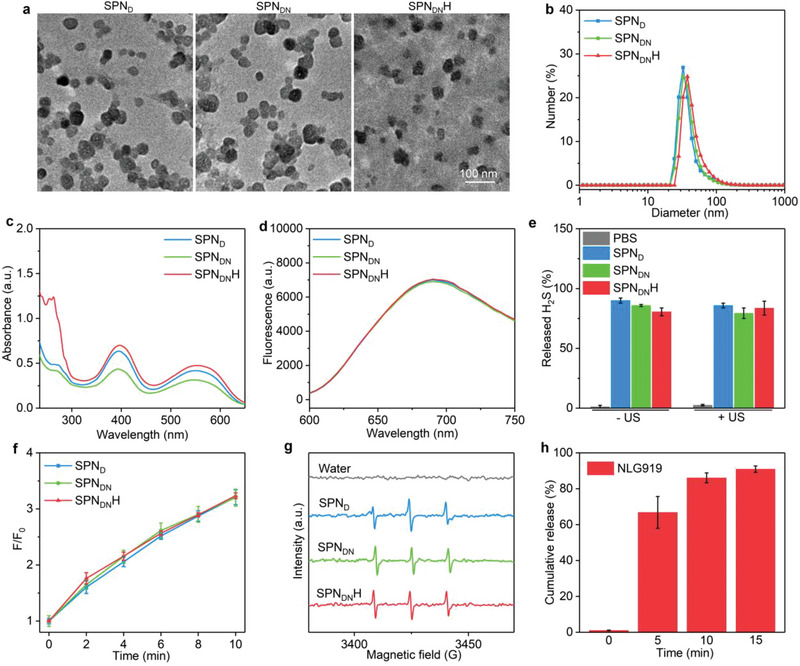
Sonodynamic and drug release properties of nanoreshapers. a) Morphology characterization of SPN_DN_H, SPN_D_, and SPN_DN_ using transmission electron microscopy (TEM). b) Diameter profiles of SPN_DN_H, SPN_D_, and SPN_DN_. c) Absorbance spectra of SPN_DN_H, SPN_D_, and SPN_DN_. d) Fluorescence emission spectra of SPN_DN_H, SPN_D_, and SPN_DN_. e) Release percentages of H_2_S from SPN_DN_H without or with US irradiation (*n* = 3). f) The ^1^O_2_ generation efficacies of SPN_DN_H, SPN_D_, and SPN_DN_ (*n* = 3). g) ESR spectra of ^1^O_2_ for SPN_DN_H, SPN_D_, SPN_DN_ and water after US treatment. h) Percentages of the released NLG919 from SPN_DN_H after US irradiation for different time (*n* = 3). Data are presented as means ± SD.

The physicochemical properties of SPN_DN_H and control counterparts (SPN_D_ and SPN_DN_) were evaluated. Approximate spherical morphologies were observed for these nanomaterials and they had a fairly homogeneous size (**Figure** **2**a). The hydrodynamic diameter of SPN_DN_H (46.4 nm) was slightly larger than that of SPN_D_ (39.5 nm) and SPN_DN_ (40.8 nm) (Figure 2b). The protein content in SPN_DN_H was observably increased compared to that in SPN_D_ and SPN_DN_ (Figure S2, Supporting Information), verifying surface HAase modification. Zeta potential was measured to be −27.6 mV for SPN_D_, −27.5 mV for SPN_DN_, and −33.6 mV for SPN_DN_H (Figure S3, Supporting Information). No noteworthy increase in hydrodynamic diameters was observed for SPN_DN_H, SPN_D_, and SPN_DN_ (Figure S4, Supporting Information), verifying the good stability. All these nanoparticles showed good hemocompatibility as no obvious hemolysis was observed (Figure S5, Supporting Information).

The optical properties including absorbance and fluorescence emission of nanoparticles were key for their SDT and fluorescence imaging, which were investigated. The absorbance peaks of PFODBT at 398 and 554 nm were similarly observed for SPN_DN_H, SPN_D_, and SPN_DN_ (Figure 2c). Another higher absorbance peak at around 280 nm was detected for SPN_DN_H than that for SPN_D_ and SPN_DN_, which should be assigned to the surface modified HAase. An obvious fluorescence emission peak at 690 nm was consistently observed for SPN_DN_H, SPN_D_, and SPN_DN_ (Figure 2d). These results verified that they displayed similar absorbance and fluorescence properties.

The sonodynamic effect and drug release properties of SPN_DN_H were then evaluated. Due to the loading of H_2_S donor, SPN_DN_H, SPN_D_, and SPN_DN_ displayed an appropriate 84.2% release percentage of H_2_S in regardless of US irradiation (Figure 2e). Using SOSG as an indicator of ^1^O_2_, the fluorescence intensities of solutions containing SPN_DN_H, SPN_D_, and SPN_DN_ became increasingly high (Figure S6, Supporting Information), confirming the generation of ^1^O_2_ via sonodynamic effect of semiconducting polymer under US irradiation. After 2, 4, 6, 8, and 10 min of US irradiation, the fluorescence intensities were similarly increased by around 1.8‐, 2.2‐, 2.6‐, 2.9‐, and 3.2‐fold for the nanoparticles (Figure 2f). The sonodynamic ^1^O_2_ generation was also evaluated using electron spin resonance (ESR). The typical ^1^O_2_ signals were observed in the ESR spectra of SPN_D_, SPN_DN_, and SPN_DN_H after US treatment and the peaks were almost identical (Figure 2g). The ^1^O_2_‐responsive shells were used to achieve the on‐demand drug release upon sono‐activation. The release percentage of NLG919 from SPN_DN_H was negligible without US irradiation, while which increased to 66.9%, 86.1%, and 91.0% after 5, 10, and 15 min of US irradiation, respectively (Figure 2h). The results suggested that the release of NLG919 could be activated by US. With US irradiation, SPN_DN_H generated ^1^O_2_ to destroy ^1^O_2_‐responsive shells, leading to sono‐activatable release of drugs.

### 2.2 In Vitro Microenvironment Modulation Evaluation

**Figure 3:**
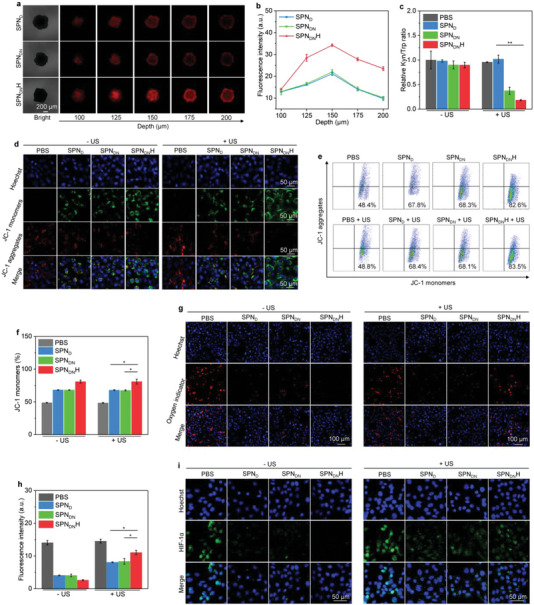
In vitro microenvironment modulation evaluation. a) Images of SPN_D_, SPN_DN_, and SPN_DN_H‐treated 3D panc02 cell spheroids at different depths. b) Fluorescence intensity analysis of SPN_D_, SPN_DN_, and SPN_DN_H‐treated 3D panc02 cell spheroids (*n* = 5). c) Kyn/Trp ratio analysis for treated panc02 cells (*n* = 5). d) Confocal fluorescence images of JC‐1 monomer and JC‐1 aggregate staining in panc02 cells. e) Flow cytometry assay of JC‐1 monomers and JC‐1 aggregates of treated panc02 cells. f) Percentages of JC‐1 monomers for treated panc02 cells (*n* = 5). g) Confocal fluorescence images of oxygen indicator in panc02 cells. h) Fluorescence intensity of oxygen indicator signals in panc02 cells (*n* = 5). i) Confocal fluorescence images of HIF‐1α staining in treated panc02 cells. Data are presented as means ± SD, and the significant differences were analyzed by two‐tailed unpaired t test, *p < 0.05, **p < 0.01.

3D panc02 cell spheroids were utilized to demonstrate the degradation of ECM for promoting the penetration of nanoparticles. At the same depth, red fluorescence signals of nanoparticles in SPN_DN_H‐treated cells were stronger than those in SPN_D_‐ and SPN_DN_‐treated cells (**Figure** **3**a). At the depth of 125, 150, 175, and 200 µm, fluorescence intensity in SPN_DN_H group was around 1.7‐, 1.6‐, 2.0‐, and 2.3‐fold higher than that in SPN_D_ and SPN_DN_ groups (Figure 3b), respectively. These results reflected that SPN_DN_H with surface modification of HAase could greatly improve nanoparticle penetration in cell spheroids.

Nanoparticle treatment with US irradiation was found to increase the expression levels of IDO in cancer cells (Figure S7, Supporting Information), which promoted the IDO‐based immunosuppression. The ability of SPN_DN_H to inhibit IDO activity was investigated by measuring the extracellular contents of kynurenine (Kyn) and tryptophan (Trp). Kyn/Trp ratio in SPN_DN_ + US and SPN_DN_H + US groups was observably reduced, which however in sole SPN_D_, SPN_DN_, and SPN_DN_H‐treated groups did not have obvious decline (Figure 3c). These results indicated that SPN_DN_ and SPN_DN_H after sono‐activation could inhibit the activity of IDO to reverse the levels of Kyn and Trp.

H_2_S production in cancer cells was confirmed using a H_2_S detection kit. Compared to PBS group, SPN_D_, SPN_DN_, and SPN_DN_H groups displayed increased H_2_S levels in cancer cells regardless of US irradiation (Figure S8, Supporting Information), verifying the H_2_S production. The disturbance of mitochondrion functions by the released H_2_S of SPN_DN_H was evaluated. Dense green fluorescence signals of 5,5',6,6‐'tetrachloro‐1,1',3,3'‐tetraethylbenzimidazoly‐carbocyanine iodide (JC‐1) monomers were detected in SPN_DN_H‐, SPN_D_‐, and SPN_DN_‐treated panc02 cancer cells regardless of US irradiation, while which were hardly observed in control cells (Figure 3d). The red fluorescence signals of JC‐1 aggregates in cells after treatments with these nanoparticles were weaker than those in PBS control cells. Flow cytometry assay showed that the percentages of JC‐1 monomers in nanoparticle‐treated cells were observably increased compared to those in PBS‐treated cells (Figure 3e). The percentages of JC‐1 monomers in SPN_DN_H + US (80.9%), SPN_DN_H – US (81.0%), SPN_DN_ + US(67.5%), SPN_DN_ – US (68.1%), SPN_D_ + US (67.9%), SPN_D_ – US (68.1%) were overall higher than those in PBS + US (48.4%) and PBS – US (48.7%) groups (Figure 3f).

After SPN_DN_H‐mediated mitochondrion function disturbance, the oxygen contents and hypoxia conditions of cancer cells were evaluated. The fluorescence signal of oxygen indicator (Ru(dpp)_3_Cl_2_) could be quenched by oxygen. The red fluorescence signals of oxygen indicator in SPN_DN_H, SPN_D_, and SPN_DN_ treated groups were weaker than those in control group (Figure 3g), suggesting the increased oxygen contents in these groups via suppressing cell respiration and oxygen consumption. The increased oxygen contents would promote the efficacy of oxygen‐dependent SDT. After SPN_DN_H, SPN_D_, and SPN_DN_ treatments plus US irradiation, the red fluorescence signals of oxygen indicator were further increased due to SDT‐mediated oxygen consumption. The fluorescence intensities of oxygen indicator in SPN_DN_H, SPN_D_, and SPN_DN_‐treated cells was at least 1.3‐fold lower than that in control cells (Figure 3h). The hypoxia conditions were verified using immunofluorescence HIF‐1α staining. Compared to PBS control group, SPN_DN_H, SPN_D_, and SPN_DN_ treatment observably reduced the fluorescence signals of HIF‐1α staining (Figure 3i). The intensities of HIF‐1α fluorescence signals in PBS + US and PBS – US groups were higher than those in SPN_DN_H, SPN_D_, and SPN_DN_ treatment groups with and without US irradiation (Figure S9, Supporting Information). The hypoxia staining signals in nanoparticle treatments plus US irradiation groups were stronger than those in nanoparticle treatments without US irradiation groups, which was because the oxygen was consumed by sonodynamic effect. Thus, these nanoparticle‐mediated H_2_S release could hinder mitochondrial functions via inhibiting cytochrome c oxidase activity, which further inhibited cell respiration and oxygen consumption, contributing to alleviation of hypoxia condition.^[^ ^20^ ^]^

### 2.3 In Vitro ICD Effect and Therapeutic Efficacy Evaluation

**Figure 4:**
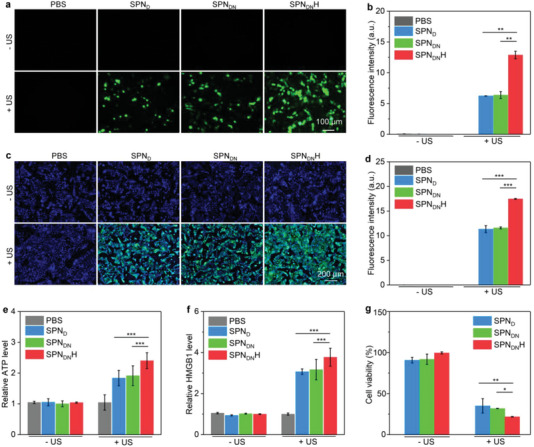
In vitro ICD effect and therapeutic efficacy evaluation. a) Fluorescence images of panc02 cells with generation of ROS. b) Fluorescence intensity of ROS signals in treated panc02 cells (*n* = 5). c) Confocal fluorescence images of CRT staining for treated panc02 cells. d) Fluorescence intensity of CRT staining signals of panc02 cells (*n* = 5). e) ATP secretion levels for treated panc02 cells (*n* = 5). f) HMGB1 secretion levels for treated panc02 cells (*n* = 5). g) Cell viability analysis of panc02 cells treated with SPN_DN_H, SPN_D_, and SPN_DN_ plus US irradiation (*n* = 5). Data are presented as means ± SD, and the significant differences were analyzed by two‐tailed unpaired *t* test, *p < 0.05, **p < 0.01, ***p < 0.001.

SDT‐mediated generation of ^1^O_2_ was confirmed by fluorescence imaging using ROS probe. ROS signals (green fluorescence) were only observed in SPN_DN_H + US, SPN_D_ + US, and SPN_DN_ + US groups (**Figure** **4**a), which suggested the generation of ^1^O_2_. Moreover, ROS fluorescence signal in SPN_DN_H + US group was stronger than that in SPN_D_ + US and SPN_DN_ + US groups. Compared to PBS group, ROS signal intensity was increased by 25.0‐, 25.5‐, and 51.6‐fold for SPN_D_ + US, SPN_DN_ + US, and SPN_DN_H + US groups, respectively (Figure 4b). These results confirmed the ^1^O_2_ generation via SDT effect.

In vitro ICD effect for nanoparticle treatment and US irradiation was investigated. Remarkable green signals were detected in CRT staining images of SPN_D_ + US, SPN_DN_ + US, and SPN_DN_H + US groups, which was much distinct from those in SPN_D_, SPN_DN_, and SPN_DN_H‐treated groups (Figure 4c). SPN_D_ + US, SPN_DN_ + US, and SPN_DN_H + US group was found to have a 11.3‐, 11.6‐, and 17.5‐fold increase in fluorescence intensity of CRT signals, respectively (Figure 4d). The treatment of SPN_D_, SPN_DN_, and SPN_DN_H plus US irradiation could increase ATP secretion levels by 1.8‐, 1.9‐, and 2.4‐fold, respectively (Figure 4e). No obvious changes in ATP secretion levels were observed for cells only after treatment of nanoparticles without US irradiation. The HMGB1 secretion levels in SPN_D_ + US, SPN_DN_ + US, and SPN_DN_H + US groups were observably increased (Figure 4f). These results manifested that SPN_DN_H‐mediated SDT effectively induced cancer cell ICD with increased expression of CRT and secretion levels of ATP and HMGB1.

The in vitro therapeutic efficacy was investigated using Panc02 cells. Panc02 cells after treatments with SPN_DN_H, SPN_D_, and SPN_DN_ at various concentrations for different times (24, 48, and 72 h) showed a cell viability higher than 90.1% (Figure S10, Supporting Information), which indicated their low cytotoxicity even after 72 h of incubation. In SPN_DN_H + US, SPN_DN_ + US, and SPN_D_ + US groups, the cell viability was reduced to 35.0%, 31.8%, and 21.7%, respectively (Figure 4g). These results confirmed the excellent in vivo therapeutic effect via SDT.

### 2.4 In Vivo Tumor Microenvironment Modulation Evaluation

**Figure 5:**
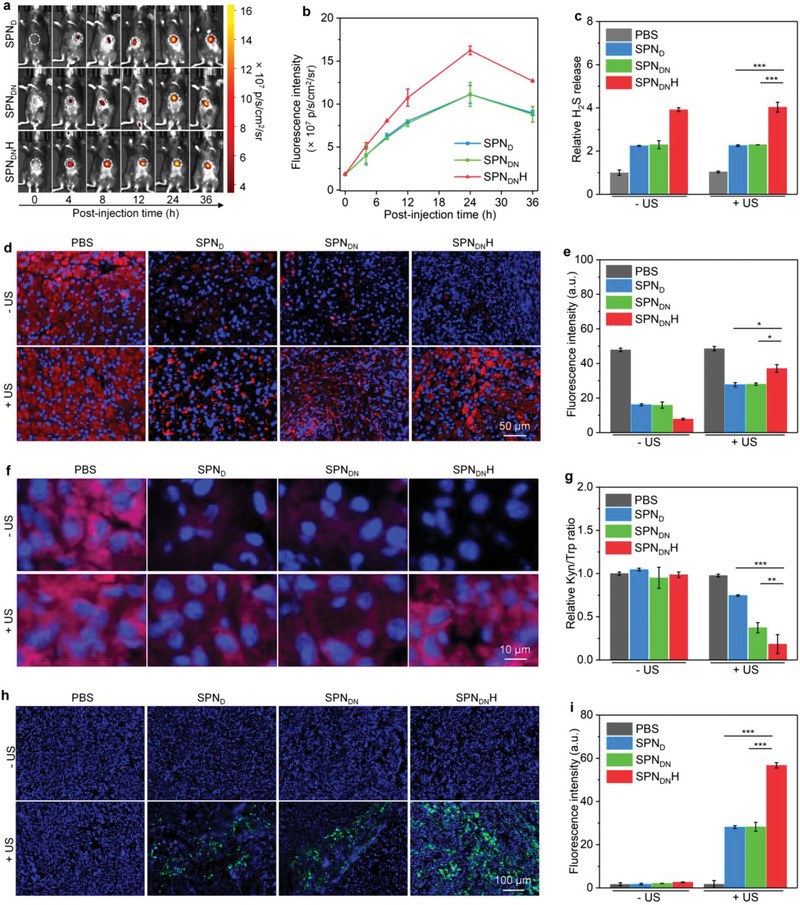
In vivo tumor microenvironment modulation evaluation. a) In vivo fluorescence imaging analysis of Panc02 orthotopic pancreatic cancer‐bearing mice after i.v. injection of SPN_D_, SPN_DN_, and SPN_DN_H. b) The fluorescence intensity of orthotopic pancreatic tumors at different post‐injection time (*n* = 3). c) Intratumoral H_2_S levels (*n* = 5). d) Confocal fluorescence images of HIF‐1α staining of orthotopic pancreatic tumors. e) Fluorescence intensity of HIF‐1α staining of orthotopic pancreatic tumors in each group (*n* = 5). f) Confocal fluorescence images of oxygen indicator in orthotopic pancreatic tumors of each group. g) Kyn/Trp ratio in orthotopic pancreatic tumors (*n* = 5). h) Confocal fluorescence images of the generated ^1^O_2_ in orthotopic pancreatic tumors of each group. i) Fluorescence intensity of generated ^1^O_2_ signals in orthotopic pancreatic tumors (*n* = 5). Data are presented as means ± SD, and the significant differences were analyzed by two‐tailed unpaired *t* test, *p < 0.05, **p < 0.01, ***p < 0.001.

The degradation of tumor ECM was first verified by measuring the contents of hyaluronic acid and accumulation of nanoparticles in tumor sites. The contents of hyaluronic acid in orthotopic pancreatic tumor tissues for SPN_DN_H – US and SPN_DN_H + US groups were much lower than those for PBS – US, PBS + US, SPN_D_ – US, SPN_D_ + US, SPN_DN_ – US, and SPN_DN_ + US groups (Figure S11, Supporting Information). This indicated that SPN_DN_H with surface conjugation of HAase could effectively degrade hyaluronic acid in the tumor ECM. The tumor fluorescence signals elevated after intravenous (i.v.) injection of SPN_D_, SPN_DN_, and SPN_DN_H and the strongest fluorescence signals could be detected at 24 h post i.v. injection (**Figure** **5**a). The intensity of fluorescence signals in tumors for SPN_DN_‐injected and SPN_D_‐injected mice was lower than that for SPN_DN_H‐injected mice (Figure 5b). Particularly, the fluorescence intensity in SPN_DN_H group was around 1.3‐fold higher than that in SPN_D_ and SPN_DN_ groups. Thus, SPN_D_, SPN_DN_, and SPN_DN_H showed an effective accumulation in orthotopic pancreatic tumor tissues via the enhanced permeability and retention (EPR) effect, which was possibly because of the surface PEG conjugation and small sizes of these nanoparticles. In addition, SPN_DN_H had an improved tumor accumulating efficacy because HAase on the surface could destroy the ECM density to promote nanoparticle diffusion and penetration. The ex vivo fluorescence images showed that SPN_D_, SPN_DN_, and SPN_DN_H mainly accumulated into tumors, livers, and spleens after i.v. injection (Figure S12, Supporting Information). The highest accumulation efficacy of nanoparticles was observed in tumors, especially for SPN_DN_H.

The intratumoral H_2_S levels were studied to verify the modulation of tumor microenvironment. The i.v. injection of SPN_D_, SPN_DN_, and SPN_DN_H could observably increase the intratumoral H_2_S levels regardless of US irradiation (Figure 5c). The H_2_S levels in SPN_DN_H‐injected group were around 1.8‐fold higher than those in SPN_D_‐ and SPN_DN_‐injected groups. This should be because the improved accumulation of SPN_DN_H in tumor tissues released more H_2_S. The hypoxia conditions in tumors after treatments were evaluated using immunofluorescence HIF‐1α staining (Figure 5d). The red fluorescence signals of HIF‐1α staining in SPN_D_, SPN_DN_, and SPN_DN_H‐treated groups were observably reduced, suggesting the effective relieving of tumor hypoxia by nanoparticles via suppressing the cell respiration and oxygen consumption. The weakest HIF‐1α staining fluorescence signal was detected in SPN_DN_H‐treated group. In SPN_D_ + US, SPN_DN_ + US, and SPN_DN_H + US groups, the HIF‐1α staining fluorescence signals were increased due to the further oxygen consumption through SDT effect. Quantitative analysis revealed that the intensity of HIF‐1α fluorescence signals in SPN_D_ + US, SPN_DN_ + US, and SPN_DN_H + US groups was reduced by 1.6‐, 1.7‐, and 1.3‐fold, respectively (Figure 5e). The tumor hypoxia was then studied using oxygen indicator. The red fluorescence signals of oxygen indicator were quenched in SPN_D_ + US, SPN_DN_ + US, and SPN_DN_H + US groups, indicating higher levels of oxygen in these groups (Figure 5f). The highest oxygen level was detected in SPN_DN_H + US group because the fluorescence signal of oxygen indicator was the weakest.

The Kyn/Trp ratio in orthotopic pancreatic tumors was studied to evaluate the modulation of IDO immunosuppressive tumor microenvironment. Kyn/Trp ratios in PBS, SPN_D_, SPN_DN_, SPN_DN_H, and PBS + US groups were almost consistent, while which in SPN_D_ + US, SPN_DN_ + US, and SPN_DN_H + US groups were reduced (Figure 5g). Particularly, SPN_DN_H + US group showed a 5.5‐ and 2.0‐fold lower Kyn/Trp ratio compared to control and SPN_DN_ + US groups, respectively. These results verified that SPN_DN_H treatment plus US irradiation greatly blocked IDO activity.

After relieving of tumor hypoxia, the enhanced SDT effect of SPN_DN_H was verified by measuring the generation of ^1^O_2_ in orthotopic pancreatic tumor sites. The ^1^O_2_ signal (green fluorescence) was observed in SPN_D_ + US, SPN_DN_ + US, and SPN_DN_H + US groups (Figure 5h). However, SPN_D_, SPN_DN_, and SPN_DN_H‐treated groups did not show fluorescence signals of ^1^O_2_, similar to control groups. The fluorescence intensity of ^1^O_2_ signals in SPN_DN_H + US group was the highest (Figure 5i). These results verified that the highest ^1^O_2_ generation efficacy could be achieved in SPN_DN_H + US group.

### 2.5 Deep‐Tissue Orthotopic Pancreatic Cancer Therapeutic and Anti‐Metastasis Efficacy Evaluation

**Figure 6:**
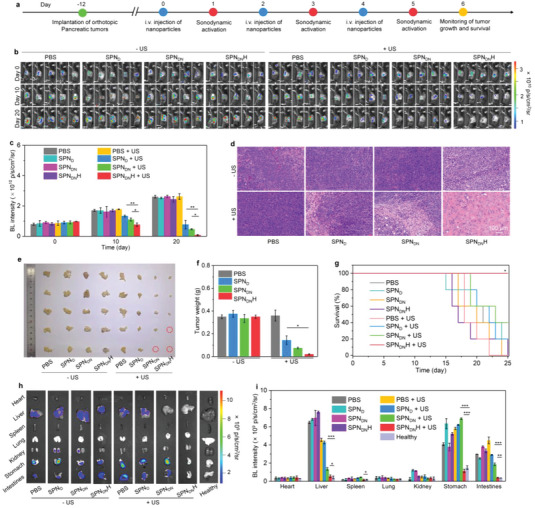
Deep‐tissue orthotopic pancreatic cancer therapeutic and anti‐metastasis efficacy evaluation. a) Schematic diagram of deep‐tissue orthotopic pancreatic cancer therapeutic efficacy evaluation via i.v. injection of SPN_D_, SPN_DN_, or SPN_DN_H and US irradiation. b) In vivo BL imaging analysis of Panc02‐Luc orthotopic pancreatic cancer‐bearing mice on day 0, 10, and 20 (*n* = 5). c) BL intensity of tumor sites for Panc02‐Luc orthotopic pancreatic cancer‐bearing mice (*n* = 5). d) Images of H&E staining of orthotopic pancreatic tumors from different treated mice. e) Photograph of tumors from orthotopic pancreatic cancer‐bearing mice (*n* = 5). f) Tumor weights of collected tumors (*n* = 5). g) Survival of Panc02‐Luc orthotopic pancreatic cancer‐bearing mice after treatments (*n* = 10). h) BL imaging analysis of tumor metastasis in different organs for Panc02‐Luc orthotopic pancreatic cancer‐bearing mice. i) BL signal intensity of intestines, stomach, kidney, lung, spleen, heart, and liver from mice in each group (*n* = 5). Data are presented as means ± SD, and the significant differences were analyzed by two‐tailed unpaired t test, *p < 0.05, **p < 0.01, ***p < 0.001.

In vivo deep‐tissue therapeutic outcomes were evaluated after i.v. injection of SPN_D_, SPN_DN_, or SPN_DN_H and US irradiation (**Figure** **6**a). Panc02‐Luc orthotopic pancreatic cancer mouse models were used to evaluate the antitumor and anti‐metastasis efficacies by measuring the bioluminescence (BL) signals. D‐Luciferin potassium salt could be oxidized by the enzyme luciferase in Panc02‐Luc cells to produce BL signals, and thus it can be used to evaluate the growths and metastasis of tumor cells. After treatment with SPN_DN_H plus US irradiation, the BL signals were gradually reduced and nearly could not be detected on day 20 (Figure 6b). Although BL signals were reduced in SPN_D_ + US and SPN_DN_ + US groups, which were still observed after treatments for 20 days. In the other groups, the BL signals increased due to the growth of tumors. The lowest BL signal intensities were always observed in SPN_DN_H + US group (Figure 6c). On day 20, the BL signal intensity in SPN_DN_H + US group was 9.6‐ and 5.8‐fold lower than that in SPN_D_ + US and SPN_DN_ + US groups, respectively.

Histological analysis showed that cell apoptosis was observed in SPN_D_ + US, SPN_DN_ + US, and SPN_DN_H + US groups (Figure 6d). Moreover, the cell apoptosis in SPN_DN_H + US group was much more severe compared to that in SPN_D_ + US and SPN_DN_ + US groups. Photographs of collected tumors showed that the sizes of tumors in SPN_D_ + US, SPN_DN_ + US, and SPN_DN_H + US groups were smaller than those of tumors in PBS group (Figure 6e). In SPN_DN_H + US group, two tumors in five individual mice were completely eradicated and the remaining three tumors showed the smallest size. The average tumor weight in SPN_DN_H + US group was as low as 0.02 g, lower than that in SPN_D_ + US (0.14 g), SPN_DN_ + US (0.08 g), and PBS (0.35 g) groups (Figure 6f). The tumor inhibition efficacy was 94.3%, 59.0%, and 78.6% for SPN_DN_H + US, SPN_D_ + US, and SPN_DN_ + US groups, respectively (Figure S13, Supporting Information). The survival of mice in SPN_DN_H + US group remained 100% after 25 days of treatment, which however was less than 50% in the other groups (Figure 6g). Thus, these nanoparticles could be utilized for treatments of deep‐tissue orthotopic pancreatic cancer due to the strong tissue penetration capability of US. The highest antitumor therapeutic efficacy was observed in SPN_DN_H + US group.

In vivo anti‐metastasis efficacy was investigated using BL imaging. Nearly no BL signals were detected in intestines, stomach, kidney, lung, spleen, heart, and liver for SPN_DN_H + US group, however the BL signals could be observed in liver, stomach and intestines in PBS, SPN_D_, SPN_DN_, SPN_DN_H, PBS + US, SPN_D_ + US, and SPN_DN_ + US groups (Figure 6h). The BL intensity of liver, stomach, and intestines for SPN_DN_H + US group was at least 2.5‐, 3.3‐, and 4.9‐fold lower than that of these organs for the other groups, respectively (Figure 6i). Moreover, the BL signals and intensities of these tissues in SPN_DN_H + US group were similar to those for healthy mice, which verified that tumor metastases were entirely suppressed.

Although activatable cancer sono‐immunotherapy was reported in a previous study, this therapeutic system only contained a semiconducting polymer and anti‐cytotoxic T‐lymphocyte‐associated protein 4 (CTLA‐4) antibody.^[^ ^21^ ^]^ Via combining SDT with immunotherapy, tumor growth inhibitions and metastasis suppression were achieved in subcutaneous 4T1 mouse breast tumors. However, orthotopic pancreatic cancer had more complicated tumor microenvironment, severe hypoxia and higher immunosuppression compared to subcutaneous 4T1 breast tumors. To multiply remodel tumor microenvironment for effective treatment of orthotopic pancreatic cancer, SPN_DN_H in this present study were designed to contain a semiconducting polymer, H_2_S donor, NLG919 and HAase, which were different from the previously reported nanosystems.

### 2.6 In Vivo ICD Effect and Immunological Effect Evaluation

**Figure 7:**
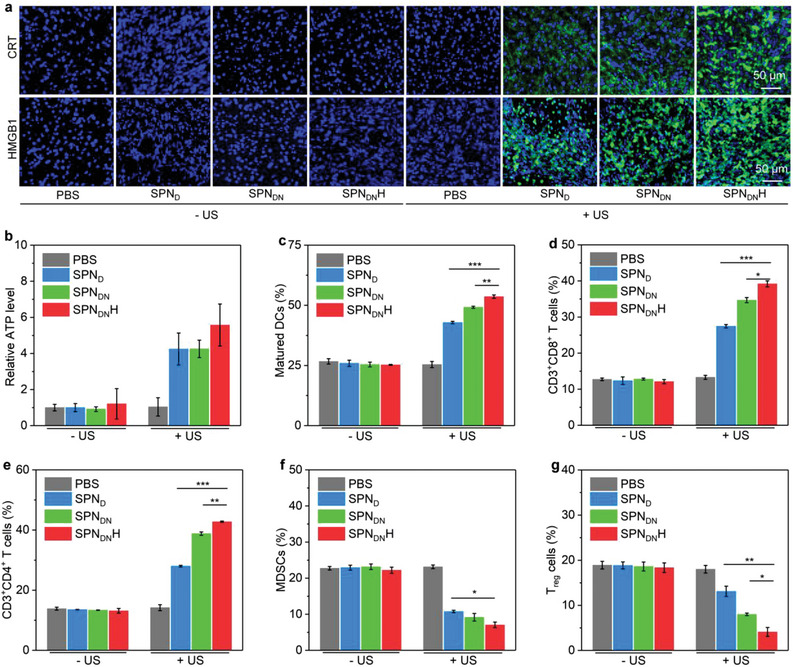
In vivo ICD effect and immunological effect evaluation. a) Confocal fluorescence images of CRT and HMGB1 staining of orthotopic pancreatic tumors. b) ATP levels in orthotopic pancreatic tumors in each group (*n* = 5). c) Contents of matured DCs (*n* = 5). d) Contents of intratumoral CD3^+^CD8^+^ T cells (*n* = 5). e) Contents of intratumoral CD3^+^CD4^+^ T cells (*n* = 5). f) Contents of intratumoral MDSCs (*n* = 5). g) Contents of intratumoral *T*_reg_ cells (*n* = 5). Data are presented as means ± SD, and the significant differences were analyzed by two‐tailed unpaired *t* test, *p < 0.05, **p < 0.01, ***p < 0.001.

Because ICD biomarkers play important roles in enhancing tumor immunogenicity, the induction of ICD effect in orthotopic pancreatic tumors was studied. Immunofluorescence analysis showed that obvious CRT and HMGB1 fluorescence signals could be detected in SPN_D_ + US, SPN_DN_ + US, and SPN_DN_H + US groups, while the signals were hardly detected in the other groups (**Figure** **7**a). SPN_DN_H + US group showed the strongest CRT and HMGB1 staining signals. The intensity of CRT fluorescence signals in SPN_DN_H + US group was increased by around 122.7‐fold compared to that in PBS group, and was 1.4‐fold higher than that in SPN_DN_ + US groups (Figure S14, Supporting Information). The highest fluorescence intensity of HMGB1 staining was also observed in SPN_DN_H + US group (Figure S15, Supporting Information). The sole treatments of SPN_D_, SPN_DN_, and SPN_DN_H did not observably increase ATP levels in orthotopic pancreatic tumors, while these nanoparticle treatments plus US irradiation increased the ATP levels by 3.5‐fold for SPN_D_ + US, 3.5‐fold for SPN_DN_ + US, and 4.6‐fold for SPN_DN_H + US groups (Figure 7b). These results verified the forceful ICD effect in SPN_DN_H + US groups.

The immunological effect in orthotopic pancreatic tumor‐bearing mice was investigated. The contents of matured DCs were only increased in SPN_D_ + US, SPN_DN_ + US, and SPN_DN_H + US groups (Figure S16, Supporting Information). The matured DC level in SPN_D_ + US, SPN_DN_ + US, and SPN_DN_H + US groups was 42.9%, 49.2%, and 53.6%, respectively, while which was around 25.0% for the remaining groups (Figure 7c). Sole treatment of SPN_D_, SPN_DN_, SPN_DN_H did not obviously affect the levels of CD3^+^CD8^+^ T cells, but nanoparticle injection with US treatment could observably increase the levels of CD3^+^CD8^+^ T cells (Figure S17, Supporting Information). CD3^+^CD8^+^ T cell level in SPN_DN_H + US group was increased to 39.2%, higher than that in SPN_D_ + US (27.5%) and SPN_DN_ + US (34.7%) groups (Figure 7d). The variation trend of CD3^+^CD4^+^ T cell levels in each group was similar to that of CD3^+^CD8^+^ T cell levels (Figure S18, Supporting Information). The highest percentage of CD3^+^CD4^+^ T cells in SPN_DN_H + US group (42.8%) was increased by 3.3‐, 1.5‐, and 1.1‐fold compared to that in control, SPN_D_ + US and SPN_DN_ + US groups, respectively (Figure 7e). The levels of immunosuppressive myeloid‐derived suppressor cells (MDSCs) in SPN_D_ + US, SPN_DN_ + US, and SPN_DN_H + US groups were found to be reduced, while which were almost the same in remaining groups (Figure S19, Supporting Information). The percentage of MDSCs was measured to be 10.8% for SPN_D_ + US, 9.2% for SPN_DN_ + US, and 7.1% for SPN_DN_H + US groups (Figure 7f). The treatments of nanoparticles with US irradiation were also found to down‐regulate the levels of regulatory T (*T*_reg_) cells (Figure S20, Supporting Information). The lowest percentage of *T*_reg_ cells in tumor tissues was observed in SPN_DN_H + US group (4.1%), which was reduced by 4.4‐fold (Figure 7g). Overall, the increased levels of DCs, CD3^+^CD4^+^, and CD3^+^CD8^+^ T cells, but reduced levels of MDSCs and *T*_reg_ cells were observed in SPN_DN_H + US group. The activation of amplified immunological effect not only greatly suppressed the orthotopic pancreatic tumor growths, but also completely restricted the tumor metastasis.

### 2.7 Side Effect Evaluation

All the orthotopic pancreatic tumor‐bearing mice showed a slightly increased body weight after various treatments (Figure S21, Supporting Information). Histological morphologies of major tissues in SPN_DN_H + US and control groups were consistent (Figure S22, Supporting Information). The values of blood routine parameters and liver/kidney function indicators in all groups were similar and remained in normal levels (Figures S23 and S24, Supporting Information). These results indicated that SPN_DN_H‐based therapeutic strategy did not cause obvious side effects.

## 3 Conclusion

In summary, we have reported sono‐activatable SPN_DN_H to precisely deliver drugs and multiply remodel tumor microenvironment for boosting antitumor immunological effect. Such SPN_DN_H were designed to contain three key reshapers to remodel the complex tumor microenvironment of orthotopic pancreatic cancer: i) HAase degraded hyaluronic acid in tumor ECM stroma, ii) H_2_S donor released H_2_S to alleviate the tumor hypoxia via inhibiting cell respiration and oxygen consumption, and iii) NLG919 blocked IDO activity to reverse the immunosuppressive tumor microenvironment. Under US irradiation, SPN_DN_H effectively generated ^1^O_2_ via the SDT effect of semiconducting polymer, which was further improved because of ECM degradation that enhanced nanoparticle tumor accumulation and hypoxia relieving. The generated ^1^O_2_ not only induced ICD effect, but also destroyed the ^1^O_2_‐responsive nanoparticle shells for sono‐activatable delivery of NLG919 into tumor tissues. The infiltration of immune cells into tumors was also improved through degradation of ECM stroma. Such a multiple remodeling strategy using SPN_DN_H triggered a forceful antitumor immunological effect, which resulted in effective growth inhibition of deep‐tissue orthotopic pancreatic tumors and resistance of tumor metastases in mouse models. This work provided an effective and precise strategy to multiply remodel tumor microenvironment for immunotherapy of deep‐seated orthotopic tumors. More efforts will be devoted to optimize the components of such sort of nanoplatforms for their applications in various types of orthotopic tumors.

## 4 Experimental Section

### Materials

Semiconducting polymer (PFODBT) was provided by Sigma‐Aldrich (USA). H_2_S content assay kit was purchased from Solarbio (Beijing, China). All solvent was provided by Sinopharm (China). IDO inhibitor (NLG919) was purchased from MedChemExpress (USA).

### Synthesis of H_2_S Donor

H_2_S donor was synthesized according to the previous report.^[^ ^19^ ^]^ Compound 1 (123 mg) was dissolved in anhydrous dichloromethane and thiophosgene (150 µL) was slowly added into the solution, and the mixed solution was stirred under nitrogen at room temperature for 30 min. The white solid (compound 2) was obtained after purification using silica gel column chromatography. 4‐Hydroxybenzaldehyde (131 mg) and dicyanoisophorone (200 mg) were co‐dissolved in ethanol and then 0.04 mL piperidine was added into the mixture and the obtained solution was refluxed for 6 h. After removal of solvent and purification via column chromatography on silica gel, compound 3 was obtained. KOH aqueous solution was mixed with tetrahydrofuran solution of compound 3 (250 mg), and the solution was stirred at room temperature for 10 min. The obtained solution was dropwise added into anhydrous tetrahydrofuran containing tiophosgene, and the solution was stirred under nitrogen at room temperature for 60 min. After purification, compound 4 was obtained. Compound 2 (36 mg), 4‐dimethylaminopyridine (53 mg) and compound 4 (80 mg) were co‐dissolved in 6 mL anhydrous dichloromethane and the solution was stirred at room temperature for 10 min. After purification via silica column chromatography, H_2_S donor was obtained.

### Synthesis of ^1^O_2_‐Responsive Polymer

A ^1^O_2_‐responsive polymer, 1,2‐distearoyl‐sn‐glycero‐3‐phosphoethanolamine‐thioketal‐(polyethylene glycol) (DSPE‐TK‐PEG) was synthesized according to the previous study.^[^ ^22^ ^]^

### Synthesis of Nanoparticles and Control Counterparts

PFODBT (0.25 mg), H_2_S donor (0.5 mg), NLG919 (0.5 mg), DSPE‐PEG‐NHS (10.0 mg), and DSPE‐TK‐PEG (10.0 mg) were dispersed in 1.0 mL tetrahydrofuran (THF) solution, respectively. Then, the above solution was mixed and quickly added into a mixture solution (*V*_THF_:*V*_water_ = 1:9) to form nanoparticles. The solvent in nanoparticle solution was removed by volatilizing in a shaker for 24 h, and then the solution was filtered. The resulting solution was dialyzed for 2 days (*M*_w_ = 10 kD) to obtain SPN_DN_. The PBS solution of SPN_DN_ was then mixed with HAase and the resulted solution was reacted at 4 °C. The nanoparticle solution was purified to obtain the final sample (SPN_DN_H). SPN_D_ (the nanoparticles loaded with PFODBT and H_2_S donor) were synthesized as the control using a similar synthesis method.

### Characterization of Nanoparticles

The diameters and surface potentials of SPN_D_, SPN_DN_, and SPN_DN_H were investigated using a Malvern Zetasizer (Nano‐ZS90). UV–vis absorptions were tested using a UV–vis spectrophotometer. The hydrodynamic diameters of samples were measured on day 0, 7, and 14 to evaluate the stability. Fluorescence properties of samples were tested using a fluorescence spectrometer.

### H_2_S Release Evaluation

The amount of H_2_S production from SPN_D_, SPN_DN_, and SPN_DN_H (concentration = 25 µg mL^−1^, 100 µL) was detected using H_2_S detection kit.

### Evaluation of Hemolysis

Mouse erythrocytes were extracted from living mice and incubated with different concentrations of SPN_D_, SPN_DN_, and SPN_DN_H for 2 h. Hemolysis rates of the erythrocytes were then calculated.

### ^1^O_2_ Production Evaluation

Singlet oxygen sensor green (SOSG) was used to confirm ^1^O_2_ production of SPN_D_, SPN_DN_, and SPN_DN_H (concentration = 15 µg mL^−1^) under US irradiation for 2, 4, 6, 8 and 10 min. The generation of ^1^O_2_ was measured using fluorescence spectrophotometer. 2,2,6,6‐Tetramethylpiperidine (TEMP, 15 µL) was added into the solutions of SPN_DN_H, SPN_D_, and SPN_DN_ (20 µg mL^−1^, 0.2 mL), and the mixed solutions were treated by US for 5 min. ESR spectrometer was used to obtain the ESR spectra of ^1^O_2_.

### Evaluation of Drug Release Efficacy

To assess the release of NLG919, SPN_DN_H solution (50 µg mL^−1^) was irradiated by US (1.0 W cm^−2^, 1.0 MHz, 50% cycle) for 0, 5, 10, and 15 min. The release of NLG919 was detected using HPLC.

### Assessment of In Vitro Cytotoxicity

Panc02 cancer cells were incubated with SPN_D_, SPN_DN_, or SPN_DN_H at different concentrations for 24, 48, and 72 h. Cell counting kit‐8 (CCK‐8) assay was used to assess the viability. Panc02 cells were incubated with SPN_D_, SPN_DN_, and SPN_DN_H (concentration = 50 µg mL^−1^) for 12 h. Then Panc02 cells were irradiated by US for 5 min, and CCK‐8 analysis was conducted to determine the viability.

### Assessment of Intracellular ROS Production

Panc02 cells were incubated with SPN_D_, SPN_DN_, and SPN_DN_H (25 µg mL^−1^) for 6 h. H_2_DCFH‐DA contained fresh DMEM medium was used for culture of cells for 30 min. After irradiation of cells with US (5 min), the intracellular ROS production was verified using fluorescence microscope.

### Assessment of Intracellular H_2_S Release

Panc02 cells were incubated with SPN_D_, SPN_DN_, and SPN_DN_H (25 µg mL^−1^) and then treated by US. The levels of H_2_S inside the treated cells were determined using H_2_S detection kit.

### Evaluation of ICD In Vitro

To assess the ICD effect in vitro, Panc02 cells were treated with SPN_D_, SPN_DN_, and SPN_DN_H (25 µg mL^−1^) with or without US irradiation (5 min). The levels of ATP and HMGB1 in cell culture medium were detected using ELISA kits, respectively. CRT expression was estimated via immunofluorescence staining.

### Evaluation of In Vitro Trp Metabolism

Trp metabolism in Panc02 cells was evaluated according to methods as reported in the previous study.^[^ ^22^ ^]^

### Evaluation of Nanoparticle Penetration in Multicellular Spheroids

To assess the penetration of nanoparticles, multicellular Panc02 spheroids were built and treated with SPN_D_, SPN_DN_, and SPN_DN_H (50 µg mL^−1^). Images of multicellular spheroids at different depths (100, 125, 150, 175 and 200 µm) were recorded by confocal fluorescence microscopy.

### Measurement of Mitochondrion Membrane Potential

Panc02 cells were treated with PBS, SPN_D_, SPN_DN_, and SPN_DN_H (25 µg mL^−1^), and irradiated by US (5 min). The cells were incubated with JC‐1 detecting agent and the expression levels of JC‐1 aggregates/monomers were measured using confocal microscopy and flow cytometer.

### The O_2_ Content and Hypoxia Detection In Vitro

Panc02 cells were incubated with PBS, SPN_D_, SPN_DN_, and SPN_DN_H (25 µg mL^−1^) and then irradiated by US (5 min). The cells were then stained by oxygen indicator and Hoechst. The O_2_ contents were evaluated using laser scanning confocal microscope. The treated cells cultured in a hypoxia chamber were used for immunofluorescence staining of hypoxia‐inducible factor 1‐α (HIF‐1α).

### Assessment of IDO Expression Levels In Vitro

Panc02 cells were treated with PBS, SPN_D_, SPN_DN_, and SPN_DN_H (25 µg mL^−1^) and US irradiation (5 min). The cells in different groups were used for evaluation of IDO expression levels using western blot (WB) assay.

### Establishment of Orthotopic Pancreatic Cancer Mouse Models

Animal experiments were allowed by the animal care and treatment committee of Donghua University (approval number: DHUEC‐NSFC‐2022‐16). Orthotopic pancreatic cancer mouse tumor models were established in C57BL/6 mice. Panc02 cell suspension (2 × 10^6^ cells, 15 µL) was injected into the pancreas of each C57BL/6 mouse.

### Evaluation of Intratumoral Hyaluronic Acid Degradation

To assess intratumoral hyaluronic acid contents, orthotopic pancreatic Panc02 tumors were collected from mice after injection of PBS, SPN_D_, SPN_DN_, or SPN_DN_H (concentration = 200 µg mL^−1^, 0.2 mL) via tail vein and ground into cell suspension in PBS solution (pH = 7.4). The contents of hyaluronic acid were measured using a mouse hyaluronic acid ELISA kit.

### In Vivo Fluorescence Imaging and Biodistribution Analysis

Orthotopic pancreatic cancer mouse models were injected with SPN_D_, SPN_DN_, or SPN_DN_H (200 µg mL^−1^, 0.2 mL) via tail vein. To study nanoparticle accumulation into orthotopic pancreatic tumors, IVIS fluorescence imaging system was used to image the mice (excitation: 520 nm, emission: 700 nm) at 0, 4, 8, 12, 24, and 36 h. The acquired images were analyzed using Living Image software. After 36 h of injection, orthotopic pancreatic cancer‐bearing C57BL/6 mice were sacrificed to obtained tissues for biodistribution analysis using IVIS fluorescence imaging system.

### Evaluation of In Vivo Antitumor and Anti‐Metastasis Efficacy

The mice with orthotopic pancreatic cancer were i.v. injected with PBS, SPN_D_, SPN_DN_, and SPN_DN_H (200 µg mL^−1^, 200 µL). At 24 h, the orthotopic pancreatic cancer were treated with US irradiation (10 min). Nanoparticle injection (on day 0, 2, and 4) and US irradiation (on day 1, 3, and 5) were repeated three times. The mice were injected with D‐luciferin potassium salt solution (20 mg mL^−1^, 150 µL) on day 0, 10, and 20. To monitor the tumor growths, IVIS imaging system was used to acquire bioluminescence (BL) images, and the images were analyzed by using Living Image software. Then various tissues were extracted from mice for ex vivo BL imaging to evaluate the anti‐metastasis effect. The orthotopic pancreatic tumors were collected to weight and photograph of tumors was obtained.

### Monitoring of Mouse Survival

The mice with orthotopic pancreatic cancer (*n* = 10) were treated with the same methods as above described and their survivals were monitored every day.

### Assessment of Intratumoral Oxygen Content and Hypoxia

Orthotopic pancreatic tumors were collected form mice after treatments as above described, and used for immunofluorescence staining of HIF‐1α. The oxygen indicator solution was intraperitoneally injected into mice in various treated groups to evaluate the intratumoral oxygen contents.

### In Vivo Detection of Intratumoral ROS Production

PBS, SPN_D_, SPN_DN_, and SPN_DN_H were injected into mice with orthotopic pancreatic cancer via tail vein. At 24 h, H_2_DCFH‐DA solution (5 µm, 100 µL) was intraperitoneally injected into mice, and the tumors were treated by US irradiation (10 min). The tumors were used for ROS level evaluation using fluorescence imaging.

### In Vivo ICD Evaluation

The treated mice were sacrificed to collect orthotopic pancreatic tumors. The contents of CRT, HMGB1, and ATP in the tumor tissues were evaluated.

### In Vivo Immune Response Evaluation

After treatments of orthotopic pancreatic cancer‐bearing mice, the tumor‐draining lymph nodes (TDLNs) and orthotopic pancreatic tumors were used to prepare single cell suspensions. The obtained single cells were stained with fluorescence dye‐labeled antibodies. Immune response was evaluated by measuring the contents of stained single cells using flow cytometry.

### In Vivo Biosafety Evaluation

The orthotopic pancreatic cancer‐bearing mice after treatments were sacrificed to collect tissues for staining. Blood biochemistry and blood routine analysis of the blood samples were conducted.

### Statistical Analysis

Mean ± SD were presented in data. The sample numbers (*n*) were provided, and significant differences were indicated as *(*p* < 0.05), **(*p* < 0.01), and ***(*p* < 0.001). A two‐tailed unpaired *t* test was adopted to determine the statistical significance. GraphPad Prism 8.0 was used for statistical analysis.

## Conflict of Interest

The authors declare no conflict of interest.

## Supporting information

Supporting Information

## Data Availability

The data that support the findings of this study are available from the corresponding author upon reasonable request.
