# Correction to “Sono‐Activatable Semiconducting Polymer Nanoreshapers Multiply Remodel Tumor Microenvironment for Potent Immunotherapy of Orthotopic Pancreatic Cancer”

**DOI:** 10.1002/advs.74511

**Published:** 2026-02-24

Meng Li, Yue Liu, Yijing Zhang, Ningyue Yu, Jingchao Li*

Advanced Science, 2023, 10, 35, 2305150

https://doi.org/10.1002/advs.202305150

The fluorescence imaging panel of the mouse in **Figure 5a** was incorrectly placed and has now been replaced with the correct image.


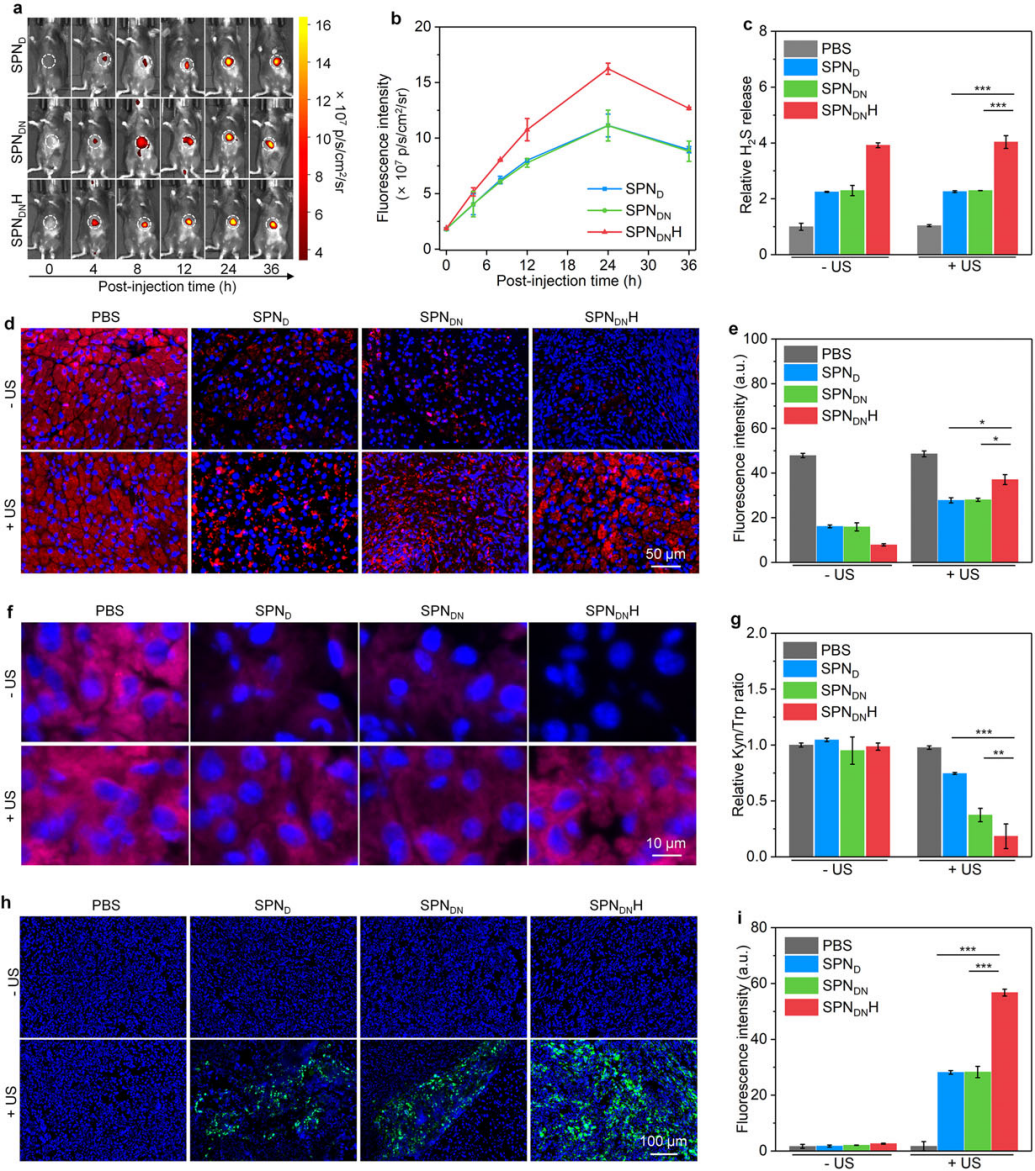


The fluorescence imaging panel of the mouse in **Figure 6b** was incorrectly placed and has now been replaced with the correct image.


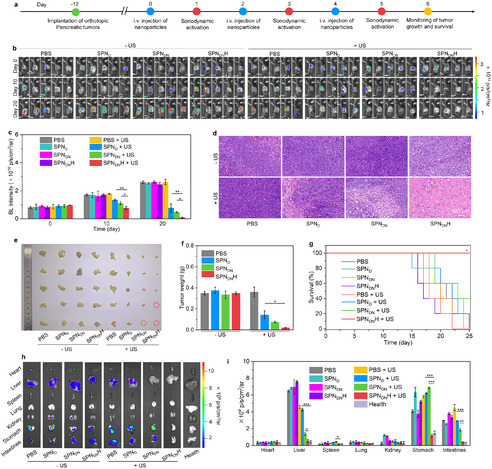


We apologize for the errors.

