# Supplementary material for: Sono‐Activatable Semiconducting Polymer Nanoreshapers Multiply Remodel Tumor Microenvironment for Potent Immunotherapy of Orthotopic Pancreatic Cancer
Source: Adv Sci (Weinh). 2023 Oct 23;10(35):2305150. doi: 10.1002/advs.202305150 (PMC10724419; doi:10.1002/advs.202305150)
Supplement: Supplementary file 1 — Supporting Information [file ADVS-10-2305150-s001.pdf]

ADVANCED SCIENCE
Open Access

Supporting Information

for *Adv. Sci.*, DOI 10.1002/advs.202305150

Sono-Activatable Semiconducting Polymer Nanoreshapers Multiply Remodel Tumor Microenvironment for Potent Immunotherapy of Orthotopic Pancreatic Cancer

*Meng Li, Yue Liu, Yijing Zhang, Ningyue Yu and Jingchao Li**

Copyright WILEY-VCH Verlag GmbH & Co. KGaA, 69469 Weinheim, Germany, 2018.

Sono-Activatable Semiconducting Polymer Nanoreshapers Multiply Remodel Tumor Microenvironment for Potent Immunotherapy of Orthotopic Pancreatic Cancer

*Meng Li, Yue Liu, Yijing Zhang, Ningyue Yu, Jingchao Li**

M. Li, Y. Liu, Y. Zhang, N. Yu, Prof. J. Li
State Key Laboratory for Modification of Chemical Fibers and Polymer Materials, College of Biological Science and Medical Engineering, Donghua University, Shanghai 201620, China
E-mail: jcli@dhu.edu.cn

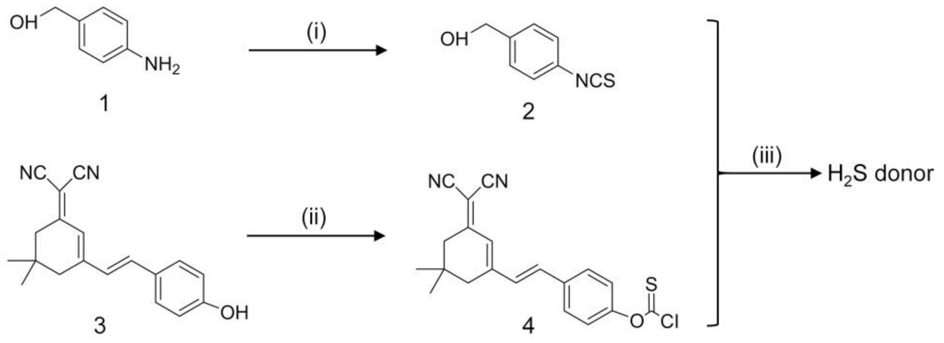

Figure S1. Synthesis route of H_2S donor. (i) Thiophosgene, dichloromethane, room temperature, 30 min; (ii) thiophosgene, tetrahydrofuran, KOH, room temperature, 60 min; (iii) 4-dimethylaminopyridine, anhydrous dichloromethane, room temperature, 10 min.

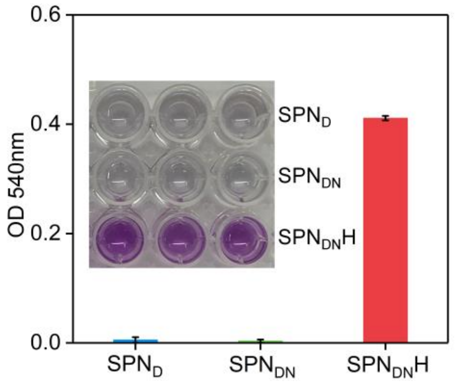

Figure S2. BCA protein assay of SPN_D, SPN_{DN} and $SPN_{DN}H$ (n = 3). Data are presented as means ± SD.

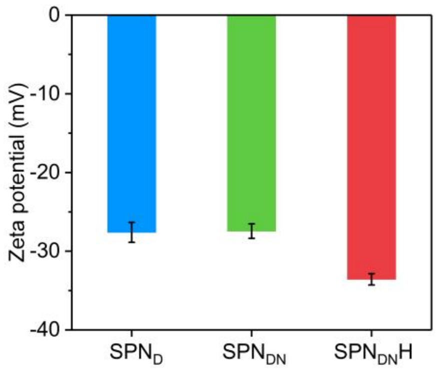

Figure S3. Zeta potential values of SPN_D, SPN_{DN} and $SPN_{DN}H$ (n = 3). Data are presented as means ± SD.

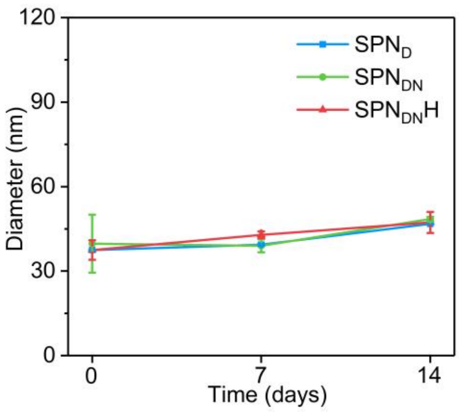

Figure S4. Hydrodynamic size measurements of SPN$_D$, SPN$_{DN}$ and SPN$_{DN}$H on day 0, 7, and 14 in water solution (n = 3). Data are presented as means ± SD.

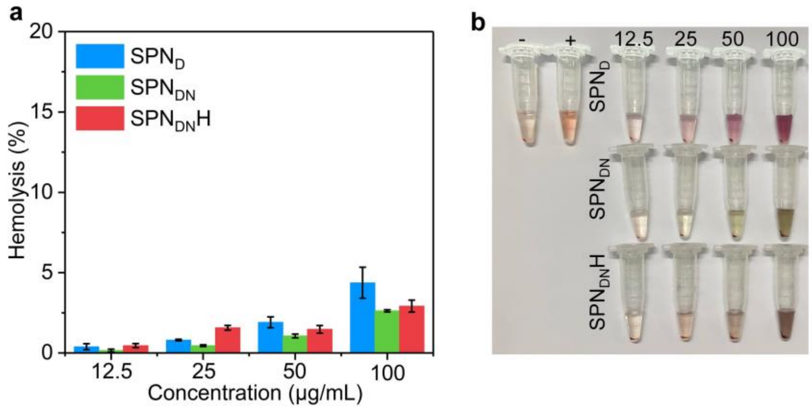

Figure S5. (a) Hemolysis percentages of murine blood red cells in different treatment groups (n = 5). (b) Hemolysis analysis of murine blood red cells after incubation with SPN$_D$, SPN$_{DN}$ and SPN$_{DN}$H at different concentrations for 2 h. Data are presented as means ± SD.

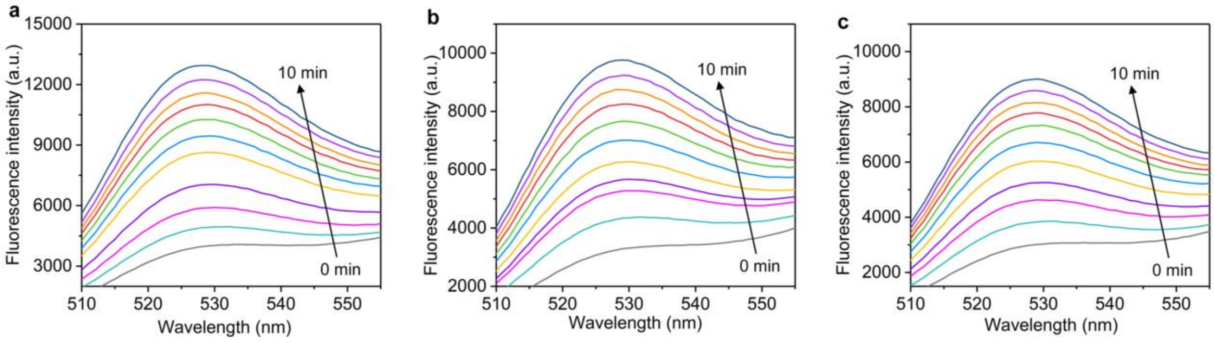

Figure S6. Fluorescence spectra of SOSG in solutions containing (a) SPN$_D$, (b) SPN$_{DN}$ and (c) SPN$_{DN}$H under US irradiation for different times.

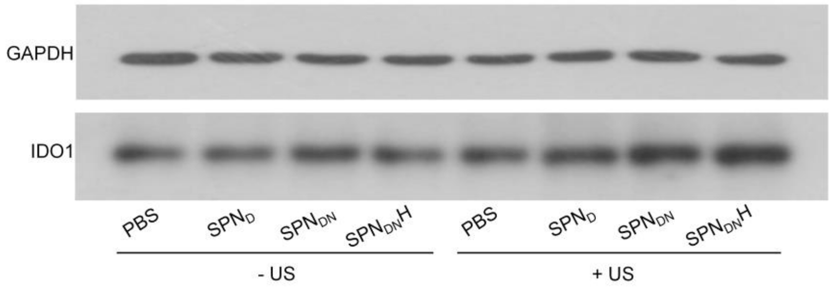

Figure S7. The WB assay of IDO expression levels in different treatment groups.

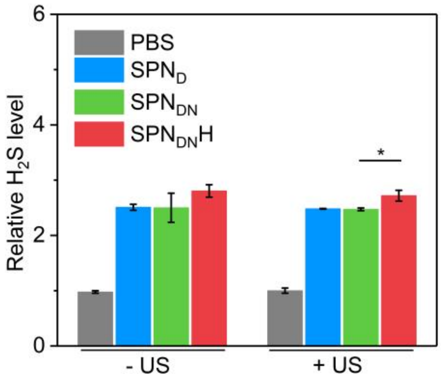

Figure S8. H_2S release levels in cancer cells after different treatments. Data are presented as means ± SD, and the significant differences were analyzed by two-tailed unpaired t test, *$p < 0.05$.

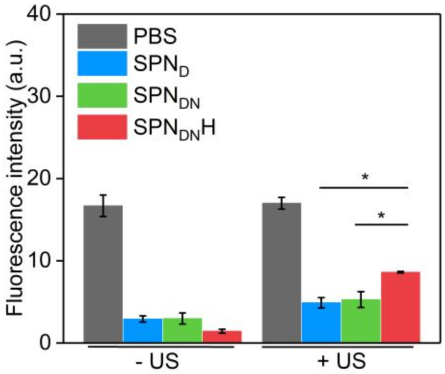

Figure S9. HIF-1α staining fluorescence intensity in various treatment groups ($n = 5$). Data are presented as means ± SD, and the significant differences were analyzed by two-tailed unpaired t test, *$p < 0.05$.

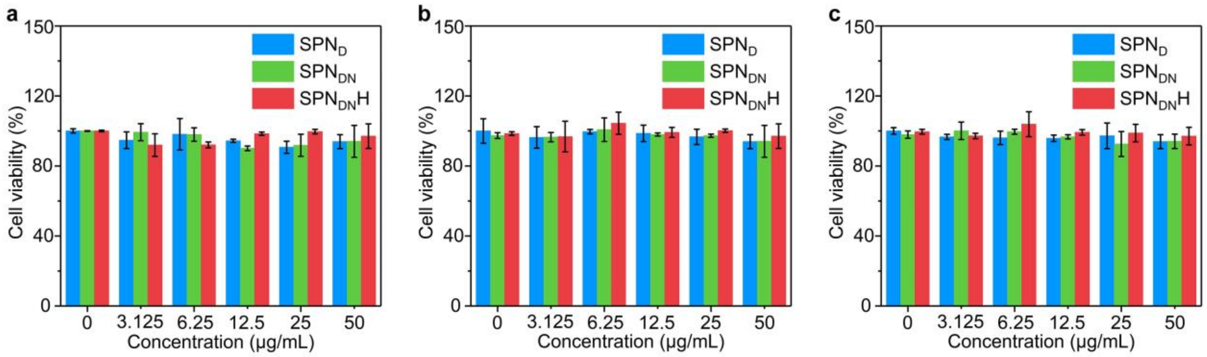

Figure S10. The cell viability of panc02 cells after incubations with SPN$_D$, SPN$_{DN}$ and SPN$_{DN}$H at different concentrations (0, 3.125, 6.25, 12.5, 25, and 50 μg/mL) for 24 h (a),48 h (b) and 72 h (c) (n = 5). Data are presented as means ± SD.

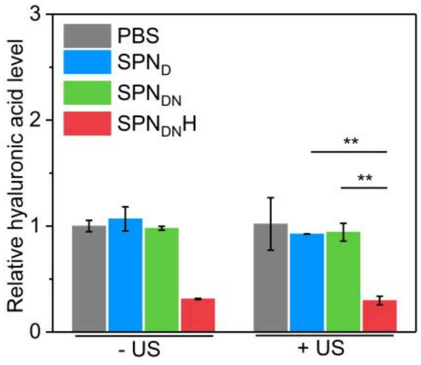

Figure S11. Hyaluronic acid levels in tumors in different treatment groups. Data are presented as means ± SD, and the significant differences were analyzed by two-tailed unpaired t test, **p < 0.01.

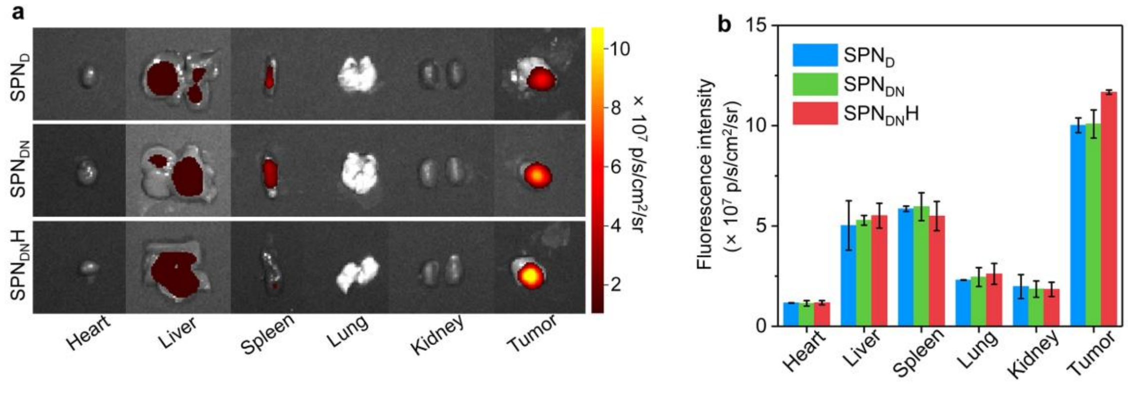

Figure S12. (a) Fluorescence images of heart, liver, spleen, lung, kidney, and tumors extracted from mice after 36 h i.v. injection of SPN$_D$, SPN$_{DN}$ and SPN$_{DN}$H. (b) Fluorescence intensity of major tissues in various groups (n = 3). Data are presented as means ± SD.

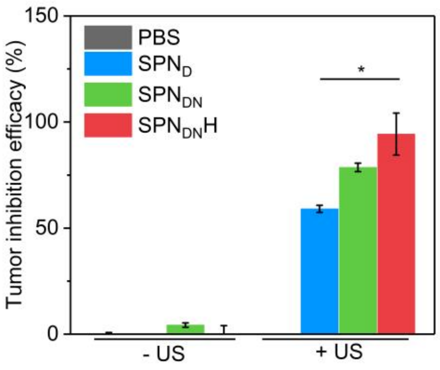

Figure S13. Tumor inhibition efficacy in different treatment groups. Data are presented as means ± SD, and the significant differences were analyzed by two-tailed unpaired t test, *p < 0.05.

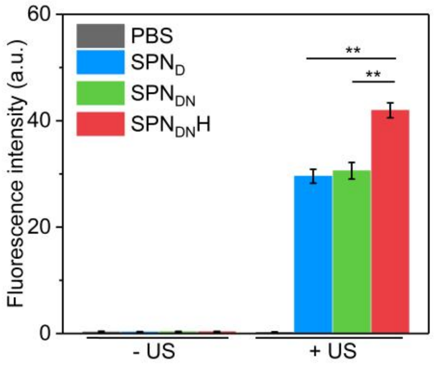

Figure S14. CRT staining fluorescence intensity of tumors from various treated mice (n = 5). Data are presented as means ± SD, and the significant differences were analyzed by two-tailed unpaired t test, **p < 0.01.

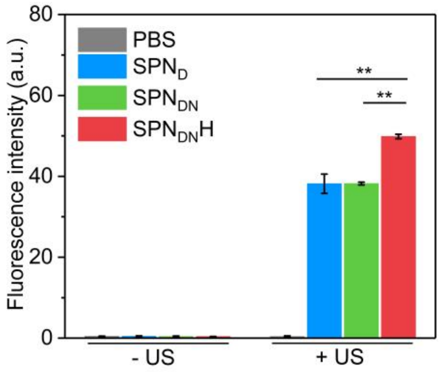

Figure S15. HMGB1 staining fluorescence intensity of tumors from various treated mice (n = 5). Data are presented as means ± SD, and the significant differences were analyzed by two-tailed unpaired t test, **p < 0.01.

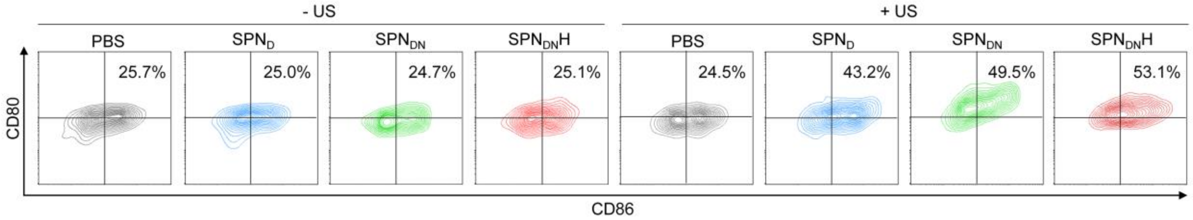

Figure S16. Flow cytometry assay of matured DCs ($CD80^+CD86^+$) in tumor draining lymph nodes in various treatment groups.

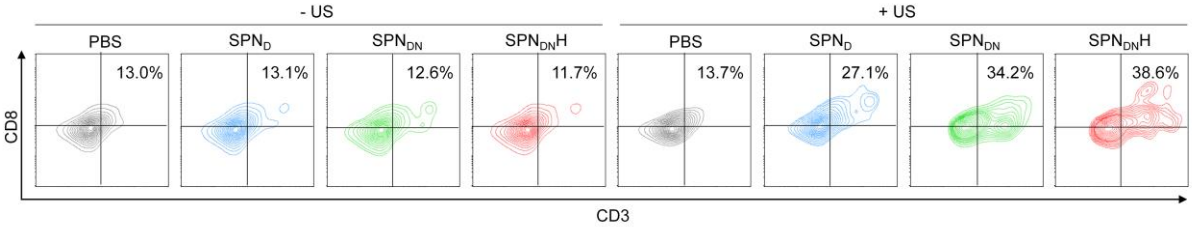

Figure S17. Flow cytometry assay of $CD3^+CD8^+$ T cells in tumors of mice with orthotopic pancreatic cancer in various treatment groups.

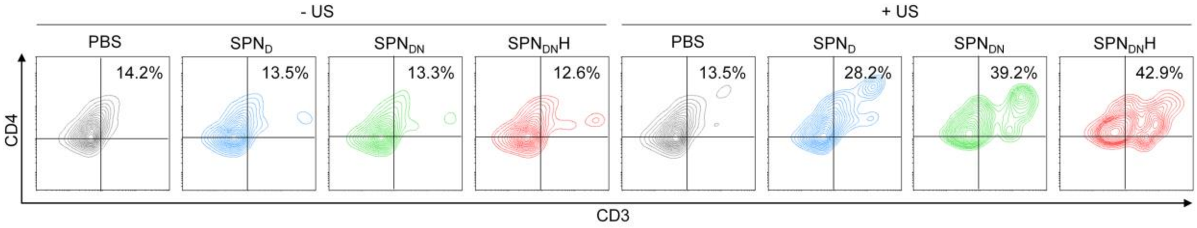

Figure S18. Flow cytometry assay of $CD3^+CD4^+$ T cells in tumors of mice with orthotopic pancreatic cancer in various treatment groups.

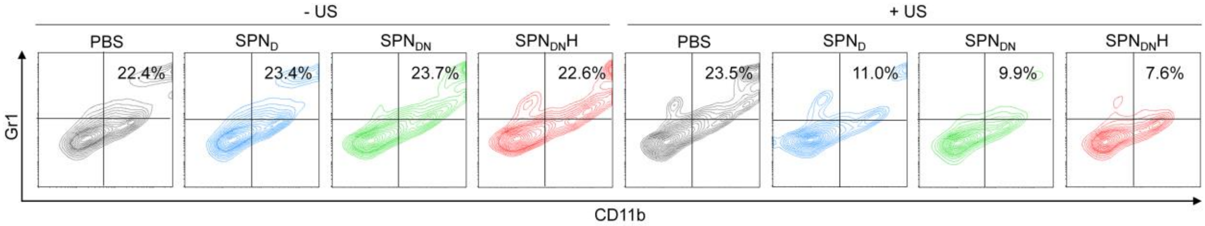

Figure S19. Flow cytometry assay of MDSCs ($CD11b^+Gr1^+$) in tumors of mice with orthotopic pancreatic cancer in various treatment groups.

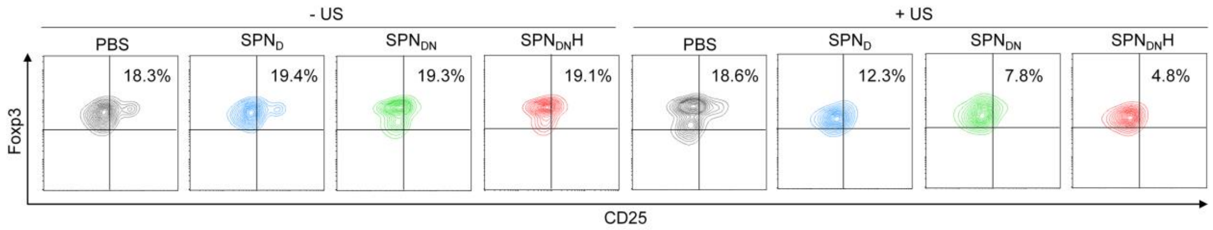

Figure S20. Flow cytometry assay of T_{reg} cells ($CD25^{+}Foxp3^{+}$) in tumors of mice with orthotopic pancreatic cancer in various treatment groups.

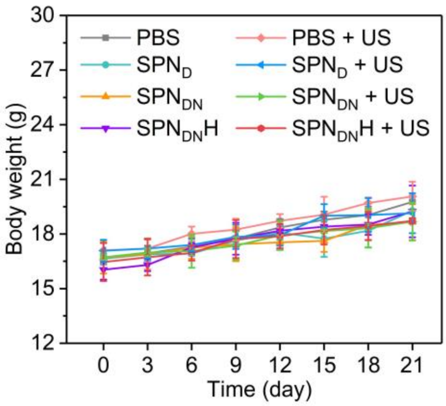

Figure S21. The body weights of mice with orthotopic pancreatic cancer in different treatment groups (n = 5). Data are presented as means ± SD.

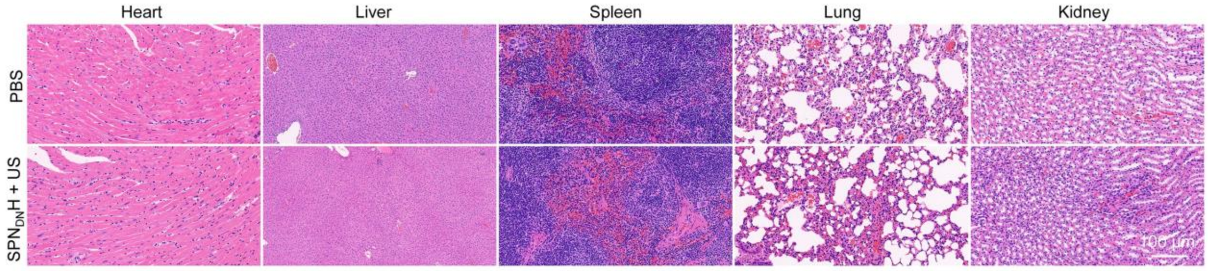

Figure S22. H&E staining images of heart, liver, spleen, lung, kidney in PBS control and $SPN_{DN}H$ + US groups after treatments for 20 days.

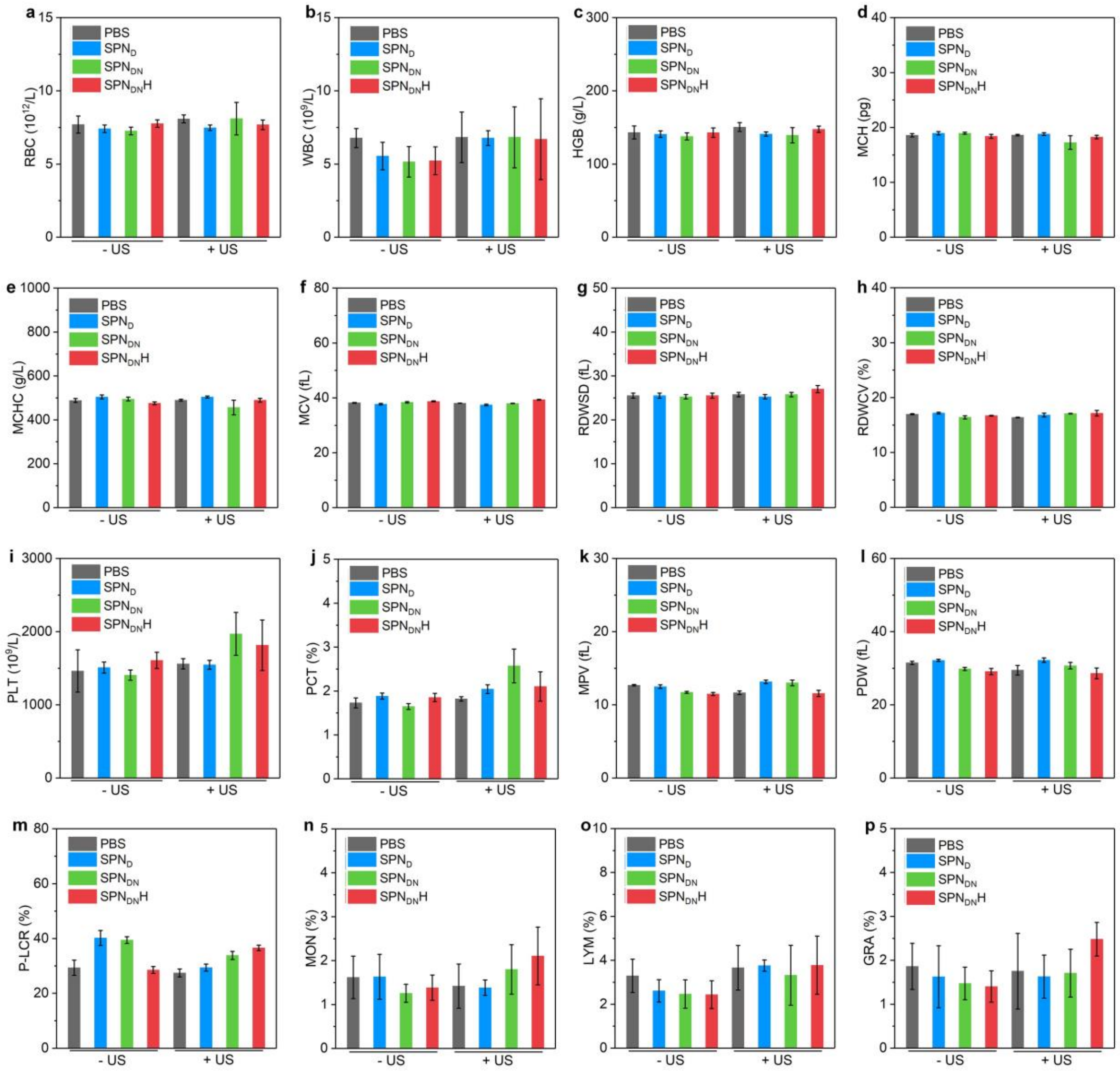

Figure S23. Blood routine analysis of (a) red blood cell count (RBC), (b) white blood cells (WBC), (c) hemoglobin (HGB), (d) mean corpuscular hemoglobin (MCH), (e) mean corpuscular hemoglobin concentration (MCHC), (f) mean corpuscular volume (MCV), (g) red blood cell distribution width standard deviation (RDWSD), (h) red blood cell distribution width coefficient of variation (RDWCV), (i) platelet (PLT), (j) plateletcrit (PCT), (k) mean platelet volume (MPV), (l) platelet distribution width (PDW), (m) platelet-larger cell ratio (P-LCR), (n) monocyte (MON), (o) lymphocyte (LYM), and (p) granulocyte (GRA) in living mice after various treatments (n = 5). Data are presented as means ± SD.

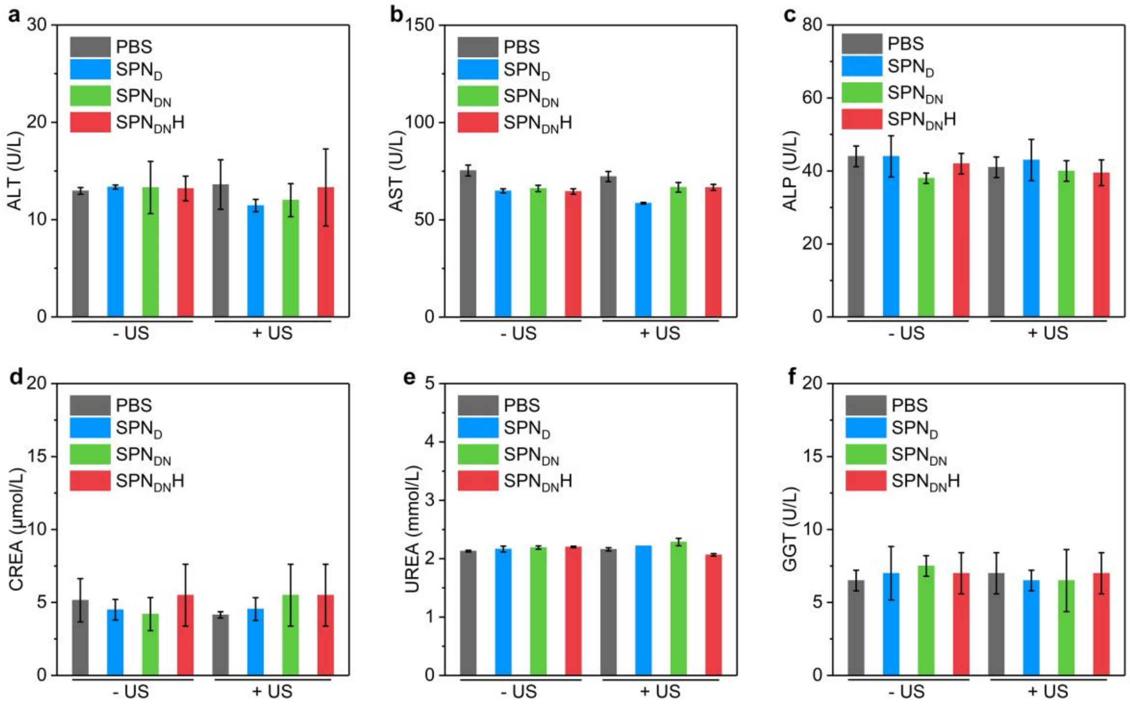

Figure S24. Blood biochemical analysis of (a) alanine aminotransferase (ALT), (b) aspartate aminotransferase (AST), (c) alkaline phosphatase (ALP), (d) creatinine (CREA), (e) urea (UREA) and (f) glutamyl transferase (GGT) in living mice after various treatments (n = 5). Data are presented as means ± SD.
